# The Moral Insignificance of Self‐consciousness

**DOI:** 10.1111/ejop.12221

**Published:** 2017-02-02

**Authors:** Joshua Shepherd

**Affiliations:** ^1^ Uehiro Centre for Practical Ethics Littlegate House, Suite 8, 16/17 St. Ebbe's Street Oxford OX11PT UK

## Abstract

In this paper, I examine the claim that self‐consciousness is highly morally significant, such that the fact that an entity is self‐conscious generates strong moral reasons against harming or killing that entity. This claim is apparently very intuitive, but I argue it is false. I consider two ways to defend this claim: one indirect, the other direct. The best‐known arguments relevant to self‐consciousness's significance take the indirect route. I examine them and argue that (a) in various ways they depend on unwarranted assumptions about self‐consciousness's functional significance, and (b) once these assumptions are undermined, motivation for these arguments dissipates. I then consider the direct route to self‐consciousness's significance, which depends on claims that self‐consciousness has intrinsic value or final value. I argue what intrinsic or final value self‐consciousness possesses is not enough to generate strong moral reasons against harming or killing.

## 1 Introduction

Many share an intuition that self‐consciousness is highly morally significant. There are various ways of explicating this significance. For some, self‐consciousness is what (morally) sets humans apart from the rest of the animal kingdom. For others, self‐consciousness is a necessary condition on possession of moral personhood, ‘full moral status’, or a right to life. In this paper, I will focus on the following explication.

*Self‐consciousness's significance*. The fact that an entity E is self‐conscious generates strong (i.e., not easily outweighed or overridden) moral reasons against harming or killing E.

I argue *Self‐consciousness's significance* is false.^1^

### 1.1 What is Self‐consciousness?

Not everyone uses the term self‐consciousness in the same way. Various phenomena can be and are lumped together by the term ‘self‐consciousness’. So it is possible that some things usefully dubbed self‐consciousness are not morally significant, and some are. I have to be careful how I use the term here.

Let us distinguish between self‐consciousness as a certain kind of capacity and self‐consciousness as a property of (phenomenal) experience.

Self‐consciousness [Capacity]. Self‐consciousness is the capacity to think of oneself as oneself, and to think of various features of oneself as features of oneself.

Self‐consciousness [Property]. Self‐consciousness is a property of phenomenal experiences: the property of for‐me‐ness.

The latter type of self‐consciousness is perhaps more difficult to understand (indeed, its existence has been challenged (cf. Prinz 2012)). Here is how Shaun Gallagher and Dan Zahavi explicate it:

There is something it is like to taste chocolate, and this is different from what it is like to remember what it is like to taste chocolate, or to smell vanilla, to run, to stand still, to feel envious, nervous, depressed or happy, or to entertain an abstract belief. Yet, at the same time, as I live through these differences, there is something experiential that is, in some sense, the same, namely, their distinct first‐personal character. All the experiences are characterized by a quality of mineness or for‐me‐ness, the fact that it is I who am having these experiences. All the experiences are given (at least tacitly) as my experiences, as experiences I am undergoing or living through. All of this suggests that first‐person experience presents me with an immediate and non‐observational access to myself, and that (phenomenal) consciousness consequently entails a (minimal) form of self‐consciousness. In short, unless a mental process is pre‐reflectively self‐conscious there will be nothing it is like to undergo the process, and it therefore cannot be a phenomenally conscious process. An implication of this is obviously that the self‐consciousness in question can be ascribed to all creatures that are phenomenally conscious, including various non‐human animals. (2015: paragraph 4)

Pre‐reflective self‐consciousness is a subtle notion. It is, in my view, far from clear that *all* experiences are ‘characterized by a quality of mineness’, even if some of them are. Additionally, it is far from clear whether this kind of self‐consciousness has moral significance (but cf. Hassoun and Kriegel 2008). In my view, this question is better explored elsewhere, as part of a discussion of the moral significance of *phenomenal* consciousness, and of the various phenomenal properties that fall under this general heading. I will not examine phenomenal consciousness here. For the kind of self‐consciousness at issue in debates about moral status is self‐consciousness [capacity]. That is the type of self‐consciousness I will examine here (henceforth, mentions of self‐consciousness will refer to this type).

### 1.2 Rationales for Self‐consciousness's Significance

Why believe that possession of self‐consciousness generates strong moral reasons against harming or killing? One might reason as follows:

There are strong moral reasons against harming or killing human beings. What grounds these reasons? Perhaps a property unique to human beings. Human beings are uniquely self‐conscious. Perhaps that's (at least part of) the grounds for the relevant moral reasons.

In addition to containing a number of questionable claims, this line of reasoning obscures our topic. Intuitions about the moral significance of human beings may track a wide range of properties, and may thus mislead us here. Even if not, we want to know about the moral significance of self‐consciousness, no matter what kind of entities possess it.

Broadly, self‐consciousness might come by its moral significance in two different ways. Call the first way *indirect*, the second *direct*. If one takes the indirect route, one argues that self‐consciousness is significant in the sense that the possession of self‐consciousness is critical (perhaps necessary, perhaps sufficient) for possession of other properties, capacities, or whatever and that these other properties, capacities, or whatever are the things that generate strong moral reasons against harming or killing. By contrast, self‐consciousness is significant in a direct way if the possession of self‐consciousness itself generates strong reasons against harming or killing.

I discuss the direct route in Section 3. Before I do, I discuss various arguments that seem to follow the indirect route.

## 2 Indirect Significance

The best‐known arguments related to self‐consciousness's moral significance take what I have called an indirect route.^2^ So self‐consciousness is argued to be necessary for possession of a crucial kind of desire (Tooley 1972), or for possession of a significant amount of future‐directed preferences (Singer 2011), or for possession of metaphysical personhood (Baker 2000). As those well‐versed in this literature will already know, all of the extant arguments relevant to self‐consciousness's moral significance have been criticized, although maybe not decisively so. What I wish to do in this section is discuss these arguments with an eye to one thing they seem to me to share. As I read them, the best‐known arguments related to self‐consciousness's moral significance share an unwarranted optimism regarding the functional significance of self‐consciousness. Once this assumption is brought into the light, much of the motivation for using these arguments to attribute moral significance to self‐consciousness dissipates. Or so I will argue.

The kind of self‐consciousness we are interested in is, as we have said, the capacity to think of oneself as oneself, and to think of various features of oneself as features of oneself. At its heart, then, the possession of self‐consciousness involves a capacity to token mental states with a certain kind of representational content—*de se* content, or content that includes explicit reference to the subject who tokens the state. For various philosophical reasons, the capacity to token such states is very interesting. But it is not clear that this capacity is functionally very important. Christopher Peacocke argues that this capacity could be widely shared throughout the animal kingdom.

The difference between the mental lives of a squirrel, an octopus, and a human does not prevent them from sharing literally the same type of *de se* content. What is common across the contents of the mental events of those very different creatures, when those events each have a *de se* content, is that each such content has a correctness condition that constitutively and fundamentally concerns the subject of the event in question. There is no ambiguity of type as between your nonconceptual *de se* and that of the octopus. They are identical in type. The differences lie rather in what the various creatures represent as holding of themselves. (2014: 14)

Peacocke draws a useful contrast between the capacity to token self‐referring states and the wealth of background knowledge a creature may deploy in representing various things as holding of themselves. A creature may possess the relevant capacity—and thus qualify as self‐conscious—without possessing a very sophisticated mental life. What is critical is not just the relevant capacity, but interactions between this capacity and a wide range of additional cognitive capacities, including abilities to represent various things as holding of oneself.

This is not to say that self‐consciousness is of no functional significance. Some have argued that the capacity to token states with de se content does come in at crucial places for cognition. Pollock and Ismael (2006), for example, have argued that this capacity is important as part of a broader operation of practical reasoning concerning the implementation of goals in unpredictable environments. Similarly, Robert Van Gulick (1988) has argued that the possession of reflexive meta‐psychological information is crucial for learning. I do not wish to disagree with either proposal here. For it is clear that far more than human beings learn—Van Gulick specifically discusses rats as an example of an animal that possesses the kind of self‐consciousness he is interested in—and it is clear that far more than human beings need to implement goals in unpredictable environments. So if these proposals are right, self‐consciousness will extend quite far into the animal kingdom.

Sometimes it is said that though non‐human animals may possess some level of self‐consciousness, their mental lives are not characterized by the kind of high‐level self‐consciousness adult humans enjoy. But we should question whether such talk is as accurate as moral theory needs it to be. For the fact is that in human beings the capacity for self‐consciousness emerges *alongside* a suite of sophisticated cognitive capacities—capacities to learn from others via imitation and application of a theory of mind, to integrate autobiographical memories, to inhibit behavioral impulses long enough to engage in practical deliberation, and so on. So it is totally unclear what kinds of functions are actually played by human self‐consciousness. Perhaps this capacity really is functionally important. Even if so, however, it is likely that its importance is strongly mediated by the development of a wide range of capacities.

If self‐consciousness is only one piece of a very complex and integrated tapestry, one wants a justification for the singling out of self‐consciousness as deserving of special attention in moral philosophy. Short of such a justification, it seems we should be discussing the significance of cognitive sophistication generally—of the whole tapestry.

Failure to appreciate the limited functional significance of self‐consciousness alone exposes moral philosophers to a danger in reasoning regarding the functional role of self‐consciousness on the one hand, and its moral significance on the other.

Consider, for example, the following passage from Peter Singer's *Practical Ethics*.

Rational, self‐conscious beings are individuals, leading lives of their own, and cannot in any sense be regarded merely as receptacles for containing a certain quantity of happiness. Beings that are conscious, but not self‐conscious, on the other hand, more nearly approximate the image of receptacles for experiences of pleasure and pain, because their preferences will be of a more immediate sort.… They will not have desires that project their images of their own existence into the future. Their conscious states are not internally linked over time. If they become unconscious, for example by falling asleep, then before the loss of consciousness they would have no expectations or desires for anything that might happen subsequently; and if they regain consciousness, they have no awareness of having previously existed. (2011: 112)

It might help Singer's case to note that since Singer suggests a wide range of non‐human animals—including many fish and birds—may be self‐conscious, he likely has relatively non‐sophisticated animals in mind here. Even so, many of the claims in this passage are questionable. Singer claims that without self‐consciousness: one's conscious states will not be ‘internally linked over time’; one will have no desires that refer to periods beyond bouts of sleep; upon awakening, one will have no awareness of one's previous existence. It is of course true by definition of any non‐self‐conscious being that its states will lack internal linkage via common reference to a concept of self; it will lack explicitly self‐referring desires; it will lack awareness of its self as a previously existing self. Maybe these kinds of claims are all Singer has in mind. But this is not what he says. Singer ignores perfectly reasonable senses in which a non‐self‐conscious entity can possess internal links between aspects of its mental life, future‐directed desires that go beyond periods of dreamless sleep, and awareness of previous episodes in its life. Without these things, it would be difficult to understand how an animal learned anything, or carried out any plans that involved components lasting longer than a few seconds.

Consider, for example, the Western Scrub Jay. This bird gives behavioral evidence of knowing what, where, and when food was stored, as well as evidence of planning for the future. Evidence in favor of the first claim stems from experiments involving the caching of two types of food—more preferred, quickly perishable wax worms and less preferred, less perishable peanuts (see Clayton and Dickinson 1998). Four hours after caching the worms and peanuts, the birds usually look for the worms first. But 124 hours after caching, the birds usually look for the peanuts instead, evincing their knowledge that the worms decay, and their memory of what was cached where, and when. Evidence in favor of the second claim stems from experiments that asked whether the birds could cache food based on expectations about future hunger as opposed to current hunger. In these experiments, the birds were trained to expect breakfast in one compartment and not another. After training, the birds were given food to cache. As the experimenters explain, ‘If they were capable of forward thinking, they should have cached more food in the compartment in which they had not been given breakfast and therefore would expect to be hungry the next morning, relative to the compartment in which they had been given breakfast’ (Raby *et al.* 2007: 919). This is what happened: a result that suggests the birds can in some sense anticipate their hunger and plan for it.

So we know that Western Scrub Jays have relatively sophisticated capacities for episodic memory, and for planning. Crucially for present purposes, however, this does not entail that they are self‐conscious. While some explanations of the birds' caching behavior would invoke self‐consciousness, alternate explanations exist. For example, it is possible that the birds' behavior is explained by their tokening of mental states such as beliefs about the past and future location of food, desires of various strengths about the past and future location of food, and by the interaction of these beliefs and desires. Importantly, on such an explanation, self‐consciousness is not required. These beliefs and desires have content about food, location, and time, and that's all. The birds need not represent their own hunger as their own in order to desire food. And they need not represent the future as their own future in order to believe that, e.g., food will be here or not there in the future.

The same lesson can be drawn from a different direction. Consider human adults with extreme cases of retrograde and anterograde amnesia. Oliver Sacks writes of one such patient, Clive Wearing, whose memory was profoundly affected by an infection of the brain. According to Sacks, Wearing was left with a memory span lasting no more than a few seconds. As a result, of course, Wearing was extremely cognitively limited. Importantly, however, his limitations were not due to self‐consciousness. Sacks describes Wearing's attempt to keep a journal:

He would write: “2:10 pm: this time properly awake.… 2:14 pm: this time finally awake.… 2:35 pm: this time completely awake,” along with negations of these statements: “At 9:40 pm I awoke for the first time, despite my previous claims.” This in turn was crossed out, followed by “I was fully conscious at 10:35 pm, and awake for the first time in many, many weeks.” This in turn was canceled out by the next entry. (2007: 187)

Obviously, Wearing was self‐conscious. What he lost were capacities for forming memories and integrating ongoing experience with the knowledge he did retain.

Singer's argumentation suggests that he conflates self‐consciousness with a suite of cognitive and behavioral capacities, and accordingly, that Singer accords self‐consciousness a certain functional significance that, as the Western Scrub Jay as well as cases of extreme amnesia illustrate, is not its due.

Is this of any moral importance? Singer follows the passage quoted above with this bit of argumentation: ‘Therefore, if they were killed while unconscious and replaced by a similar number of other members of their species who will be created only if the first group are killed, there would, from the perspective of their awareness, be no difference between that and the same animals losing and regaining consciousness’ (2011: 112). Singer's intuition about replaceability depends on claims about self‐consciousness that he takes to support claims about the particularity of an entity's mental life. Without self‐consciousness, we are told that there is nothing importantly unique about an entity's mental life (nothing worth keeping ‘from the perspective of their awareness’). But there is no good reason to think that the particularity of an entity's mental life depends upon possession of self‐consciousness. On the contrary, it is very plausible that the particularities of an entity's ongoing mental life will be structured in part by past experiences.

Because of assumptions about the functional significance of self‐consciousness, Singer is inclined to consider the lives of non‐self‐conscious beings as of no intrinsic moral significance: such beings are in essence receptacles for (very simplistic) positive and negative experiences. This is a mistake. In order to properly examine the moral significance of some feature or property of an entity, we need to get a grip on the feature or property. We need to know what it is we are examining. It is at least arguable that the greater degree of cognitive sophistication human adults (purportedly) enjoy is morally significant, irrespective of the moral significance of self‐consciousness. But that is a possibility that deserves philosophical attention separate from our current concern. So it is important not to conflate self‐consciousness with cognitive sophistication.

This point is relevant to another family of arguments that involve crucial reference to future‐directed desires. Perhaps the best‐known argument from this family is Michael Tooley's, offered in support of what Tooley calls the self‐consciousness requirement:

An organism possesses a serious right to life only if it possesses the concept of self as a continuing subject of experiences and other mental states, and believes that it is itself such a continuing entity. (Tooley 1983: 44)

Here is how Tooley initially argues for this claim. First, he asserts that the only individuals that possess rights are conscious individuals. Second, he asserts that the particular rights individuals possess are tied to their particular desires: ‘A has a right to X’ is roughly synonymous with ‘A is the sort of thing that is a subject of experiences and other mental states, A is capable of desiring X, and if A does desire X, then others are under a prima facie obligation to refrain from actions that would deprive him of it’ (45). Third, he applies this concept of rights‐possession to a right to life, which he takes to be the right to continue to exist as a subject of experiences and other mental states. So if an individual desires to continue to live in this way, then that individual is a person—they have a right to life. Since an individual can only possess this desire if they possess a self‐concept, self‐consciousness is a necessary condition on personhood.

This is a coherent argument that, if sound, could stand in defense of *Self‐consciousness's significance*. The problem is that this argument is not sound, as many commentators have convincingly argued (e.g., Marquis 1989), and as Tooley himself recognized in his (1983). There is little reason to believe that the only rights we have stem from our actual desires—a stubborn four‐year‐old who desires only candy has a right to adequate nutrition. So the second step is dubious. Without this step, Tooley's argument collapses.

Presently, however, I am less interested in Tooley's premises, and more interested in the thinking lying behind them. If one really thinks that desires create rights, then why appeal to self‐consciousness? Why not simply appeal to the wide range of existence‐entailing desires—desires to do and receive things in the near‐to‐middle‐distant future—many animals (and certainly humans) possess? One plausible diagnosis for Tooley's appeal to self‐consciousness is that he overrates the functional importance of self‐consciousness. Perhaps the thought is that without self‐consciousness, an entity could not be functionally sophisticated enough to form existence‐entailing desires.

Whether or not this kind of thinking lies behind Tooley's argumentation, it seems to lie behind a recent attempt to resuscitate it. In a recent (highly controversial) article arguing for the moral permissibility of after‐birth abortion, Alberto Giubilini and Franscesca Minerva (2013) cite Tooley approvingly in support of the claim that in order for an entity to be able to suffer harm, she would have to be mentally developed enough ‘to value the different situation she would have found herself in if she had not been harmed’ (262). They go on to claim that newborn human beings are not mentally developed enough.

Those who are only capable of experiencing pain and pleasure (like perhaps fetuses and certainly newborns) have a right not to be inflicted pain. If, in addition to experiencing pain and pleasure, an individual is capable of making any aims (like actual human and non‐human persons), she is harmed if she is prevented from accomplishing her aims by being killed. Now, hardly can a newborn be said to have aims, as the future we imagine for it is merely a projection of our minds on its potential lives. (262)

The claim about newborns lacking aims is strikingly unsupported by evidence, and seems to me at the very least extremely implausible given what we know about a normal newborn's neurocognitive development and capacities for visuocognitive behavior and learning (cf. Lagercrantz and Changeux 2009). The present point, however, is that Giubilini and Minerva explicitly link Tooley's argument for the self‐consciousness requirement with a further argument that depends on claims about a human infant's lack of functional sophistication. A claim about moral significance depends on unwarranted functional assumptions about the possession (or absence) of self‐consciousness.

In this connection, another argument in the neighborhood of Tooley's deserves discussion. Peter Singer reasons—on behalf of the preference utilitarian—as follows:

For preference utilitarians, taking the life of a person will normally be worse than taking the life of some other being, because persons are highly future‐oriented in their preferences. To kill a person is therefore, normally, to violate not just one but a wide range of the most central and significant preferences a being can have.… In contrast, beings that cannot see themselves as beings with a future do not have any preferences about their own future existence. This is not to deny that such beings might struggle against a situation in which their lives are in danger, as a fish struggles to get free of the barbed hook in its mouth; but this indicates no more than a preference for the cessation of a state of affairs that causes pain or fear. The behaviour of a fish on a hook suggests a reason for not killing fish by that method but does not in itself suggest a preference utilitarian reason against killing fish by a method that brings about death instantly, without first causing pain or distress. Struggles against danger and pain do not suggest that fish are capable of preferring their own future existence to non‐existence. (2011: 80)

Whatever one thinks about this argument,^3^ it is not clear that it requires self‐consciousness. Certainly Singer gives the impression that the argument does require self‐consciousness. He is here reasoning explicitly about why it might be wrong to kill a person, where personhood (Singer assumes) requires self‐consciousness. Further, Singer's reasoning seems to rely on self‐consciousness in a crucial place—Singer invokes the notion of a being having a concept of its own future existence in order to explain why it might be permissible (on preference utilitarian grounds) to kill a fish painlessly.

But Singer is here explicitly concerned with interests about the distant future. For a preference utilitarian, interests (or preferences) are all that matter, and the more future‐oriented you are, the more interests you might have (if you think imagining the future allows for the development of more interests). But interests about the future do not require self‐consciousness. Interests about the future can be interests *about the future*, whether or not they are about your own future existence. Consider again the Western Scrub Jay. One possible explanation of its behavior makes reference to first‐order mental states alone. Some of these mental states concern the future—the Western Scrub Jay believes that there will be no breakfast in a certain location, it desires breakfast at a future time, and this motivates it to cache food there if given the opportunity. As I noted earlier, the Western Scrub Jay may be self‐conscious—that issue remains controversial—but self‐consciousness is not necessary to explain its behavior. The same will be true of other non‐human animals who give some evidence of behavior sensitive to future contingencies. Whatever the force of Singer's argument, then, it can get by without self‐consciousness.

The arguments considered thus far depend on unwarranted assumptions about the functional importance of self‐consciousness. The final argument I wish to consider in this section makes a similar, but ultimately more subtle, mistake. I pick up on this thought after discussing the argument, which stems from work on the metaphysics of personhood.

According to Lynn Rudder Baker, a person is an irreducible ontological kind (a person has its ‘primary‐kind properties essentially’ (2005: 28)). What marks a person out as a person? ‘What distinguishes *person* from other primary kinds (like *planet* or *organism*) is that persons have first‐person perspectives’ (28). And the possession of a first‐person perspective is more or less equivalent to the possession of the capacity to think of oneself as oneself, and to think of various features of oneself as features of oneself. In Baker's words, ‘to have a first‐person perspective is to be able to think of oneself without the use of any name, description, or demonstrative; it is the ability to conceive of oneself as oneself, from the inside, as it were’ (28).

If this is right, self‐consciousness is necessary for a candidate property—metaphysical personhood—that might generate strong moral reasons against harming or killing (and this is clearly how Baker sees the moral significance of self‐consciousness; for Baker, ‘there can be no “right to life” until there is a [metaphysical] person to be a subject of that right’ (2005: 45)). But, as Baker recognizes, the account as it stands runs into problems. For there is a strong intuition that some non‐self‐conscious entities—in particular, human infants and the severely cognitively disabled—are persons. So there is a choice to be made. Leave infants and the severely cognitively disabled out of the account, or accommodate the intuition somehow. Baker takes the latter route.

For Baker, persons come into existence earlier in development than the acquisition of a first‐person perspective. At least regarding developing human beings, a person comes into existence when the human organism is sophisticated enough to support what she calls a *rudimentary* first‐person perspective.

Rudimentary FPP. A being has a rudimentary first‐person perspective if and only if (i) it is conscious, a sentient being; (ii) it has a capacity to imitate; and (iii) its behavior is explainable only by attribution of beliefs, desires, and intentions. (30)

The relevant question for present purposes is whether taking this route makes self‐consciousness unnecessary for metaphysical personhood. Baker argues not. According to Baker, the possession of a rudimentary first‐person perspective is not, on its own, significant with respect to personhood. Baker admits that some non‐human animals possess this perspective, and yet fail to qualify as persons. For Baker, what is important is that, at least in the human case, ‘the human infant's rudimentary first‐person perspective is a developmental preliminary to having a robust first‐person perspective.…’ That is, Baker maintains that not all rudimentary first‐person perspectives are metaphysically alike: ‘I mean to pick out those rudimentary first‐person perspectives that developmentally ground or underpin robust first‐person perspectives’ (33).

To review, a person is an entity that has at least a rudimentary first‐person perspective, so long as this perspective is a developmental preliminary to a robust first‐person perspective, i.e., to self‐consciousness.

Here is one place where Baker's attempt to ground personhood in self‐consciousness runs into trouble. Many non‐human animals have the capacities that constitute a rudimentary first‐person perspective. Baker admits as much. And yet these animals do not go on to develop self‐consciousness. At the very least, then, it is not only a rudimentary first‐person perspective that underpins self‐consciousness. Other things are needed—perhaps greater working memory capacity, greater attentional capacities, more sophisticated mechanisms of cognitive control and metacognition, and so on.

Though it is dubious that a rudimentary first‐person perspective *alone* developmentally underpins the development of a robust first‐person perspective, perhaps Baker would argue that this perspective at least plays some developmental role for human beings. But if the role is only partial, why single out a rudimentary first‐person perspective? What is metaphysically special about capacities for sentience, imitation, and behavior interpretable as driven by beliefs and desires? Here is Baker:

The properties in terms of which rudimentary first‐person perspectives are specified are ones we recognize as personal: sentience, capacity to imitate, intentionality. Insofar as we think of nonhuman animals as person‐like, it is precisely because they have these properties. The properties that an early‐term human fetus has – say, having a heart – are not particularly associated with persons, or even with human animals. Even invertebrates have hearts. So, not just every property that is a developmental preliminary to a robust first‐person perspective in humans contributes to being a person. There is a difference between those properties in virtue of which beings are person‐like (the properties of rudimentary first‐person perspectives) and the broader class of biological properties shared by members of many taxa. The properties in virtue of which something is a person are themselves specifically personal properties. (35)

If this is the rationale for singling out these properties, one wonders why Baker does not simply make the rudimentary first‐person perspective the distinguishing characteristic of personhood. Perhaps she does not want to include all the non‐human animals that share this perspective in the class of persons. But this is not a good reason to restrict the account to entities that will go on to possess a robust first‐person perspective. It looks, then, like Baker's account of persons suffers from an awkward tension. Either she should extend personhood to all entities that possess a rudimentary first‐person perspective or she should not appeal to this perspective as importantly different from the other developmental precursors of a robust first‐person perspective. Both options carry with them what some may see as undesirable consequences. On the former, lots of non‐human animals qualify as persons. On the latter, there is little reason to say, as Baker wishes to, that personhood emerges around the time of birth (cf. DeGrazia 2002).

In the end, Baker seems to retract her claims about the importance of development. She does so because of an objection to her view: it looks like, if the potential to develop a robust first‐person perspective is essential, then some cognitively disabled individuals will not qualify as persons. Baker wants to avoid this; as a result, she ends up making something like species membership (Baker herself talks of ‘kind’ membership) critical for personhood: ‘A being with a rudimentary first‐person perspective is a person only if it is of a kind that normally develops robust first‐person perspectives’ (33).

This view is coherent. But it is not very plausible. What Baker needs is a reason to give rudimentary first‐person perspectives person‐making significance. According to these perspectives, developmental significance does not work, since it is dubious that this perspective is really doing the relevant developmental work. Moving to kind membership does not remove the problem either—it simply buries it. Membership in a kind has little to do with the rudimentary first‐person perspective, which is now starting to look like an arbitrary feature of many kinds in the animal kingdom.

We do not find, in Baker's account, illumination regarding the purported moral significance of self‐consciousness. Baker's attempt to ground the metaphysical importance of a rudimentary first‐person perspective (which does not involve self‐consciousness) in the possession of a full‐blown first‐person perspective (which does involve self‐consciousness) falls flat.^4^

How does this relate to my earlier points regarding the function of self‐consciousness? I think assumptions about the function of self‐consciousness lie at a deeper level in Baker's thinking. As we have seen, Baker acknowledges a strong intuition that self‐consciousness undergirds personhood, as well as a strong intuition that some non‐self‐conscious entities are persons. There is an apparent conflict here, and Baker attempts to resolve these intuitions (initially) by arguing that the non‐self‐conscious entities are in some developmental sense on the way to self‐consciousness, and are persons in virtue of this.

One problem here, as I have noted, is in Baker's attempt to isolate features in virtue of which non‐self‐conscious entities are on the way to self‐consciousness. A deeper problem, I think, is that we have no great reason to conceive of an entity as developmentally on the way to self‐consciousness unless self‐consciousness is of critical functional importance for the entity. Once we see self‐consciousness as a minor part of a general trend toward cognitive sophistication, theories of personhood that include strong appeals to self‐consciousness look to suffer from misplaced focus.

In this section, I have tried to make plain ways in which many arguments relevant to the moral significance of self‐consciousness depend on unwarranted assumptions about the functional importance of self‐consciousness. Once these unwarranted assumptions are brought to light, we find nothing in these arguments that should incline us to support *Self‐consciousness's significance*. Perhaps we would do better with a more direct route.

## 3 Direct Significance

On a traditional construal, something has intrinsic moral significance (or value) if its value in some sense depends or supervenes on its internal or non‐relational properties (cf. Moore 1903). Why believe self‐consciousness has this kind of significance?

It is useful here to think about how other claims regarding intrinsic moral significance are supported. Intrinsic moral significance (or alternatively, intrinsic value) is sometimes attributed to things such as pleasurable or painful experiences, the possession of phenomenal consciousness, life, knowledge, beauty, and more (cf. Frankena 1973: 87–88). Such attributions are controversial, of course. What supports such attributions?

I submit that the best kind of support for an attribution of intrinsic moral significance to some property or capacity is that the property or capacity passes a kind of isolation test. In order to pass the test, the one administering it first needs an adequate understanding of the nature of the property or capacity. Second, one considers the property or capacity under appropriate isolation. So considered, does the thing retain moral significance? The kind of answer we need is one that is fairly clear and obvious, and one that compels widespread agreement among those that have an adequate understanding of the nature of the capacity or property.

What kind of isolation counts as appropriate?^5^ The basic procedure involves a kind of comparative test performed in reflection. One takes the property or capacity in question and considers its presence or absence in situations that hold fixed other relevant properties or capacities. This can be tricky. One wants to avoid illicitly building in features that make the thing instrumentally or extrinsically morally significant. For such features might cloud judgment regarding the thing's intrinsic significance.

Perhaps an example will help. Charles Siewert (1998) is concerned to isolate the purportedly intrinsically valuable nature of phenomenally conscious experience. Toward this end, he considers a number of ways to isolate phenomenal consciousness, at one point hitting on this way.

Suppose you thought you faced the choice between (a) continuing on leading the sort of phenomenally conscious life you expect to live, or (b) undergoing a radical phenol‐ectomy, which will make you permanently unable to have conscious experience, but will leave you (or your body) in possession of those features, for which sake you ordinarily value possession of phenomenal features: thus the nonphenomenal benefits will be the same on either option. And suppose you set aside any concerns having to do with the risks that the procedure may not work as planned. Then, if you still prefer the retention of at least *some* of them (you think (a)—the consciousness plan—is better than (b)—zombification), you do value having phenomenal features for its own sake. On the other hand, if you find that you would be *indifferent* to (or even *prefer*) the total loss of consciousness, when convinced that this would lose you none of the nonphenomenal benefits you assume would come with consciousness, then you find that you do not value consciousness for its own sake after all. (320)

Siewert uses an isolation procedure to establish that we value certain aspects of phenomenal consciousness for their own sakes, or intrinsically. If we accept that in so valuing them we are not horribly mistaken, we can accept that some aspects of phenomenal consciousness are intrinsically valuable.

How would this go with respect to self‐consciousness? In constructing an isolation test for self‐consciousness, we have to guard against the illicit introduction of extrinsically significant features. As we have already seen, self‐consciousness is sometimes associated with cognitive sophistication, and is often thought to be instrumentally significant for cognitive sophistication. In addition, self‐consciousness is sometimes associated with the enjoyment of certain types of phenomenally conscious experience (experiences of, e.g., one's life having meaning). One way to make sure we don't mistake these features for self‐consciousness's intrinsic significance is simply to rule them out.

Consider, then, two entities with relatively simple mental lives. These entities have low working memory capacity, retain little information in short‐ or long‐term memory, have fairly crude attentional capacities, fairly low‐resolution perceptual capacities, and a limited behavioral repertoire. Suppose as well that these entities are not phenomenally conscious.^6^ Suppose that the only difference between these entities is that one has self‐consciousness—it has the capacity to token mental states with self‐referring content. This entity can think of itself as itself, perhaps by occasionally thinking of its fleeting perceptual states as its perceptual states.

Such entities are conceivable. Is self‐consciousness of great moral significance for the entity that has it? Is self‐consciousness so significant that its possession generates strong reasons against harming or killing the entity that has it—reasons that are absent in the case of the otherwise similar but non‐self‐conscious entity? Plausibly, the answer to all of these questions is no. Self‐consciousness does not appear to have much intrinsic moral significance.

There is, however, another way to take the direct route. It involves claiming that though self‐consciousness lacks intrinsic moral significance, it possesses final moral significance (or final value). As Christine Korsgaard (1983) explicates final value, it is clear that understanding a thing's final value sometimes requires getting the circumstances surrounding the thing just right. This is because we ascribe (or ought to ascribe) final value to a capacity, object, or whatever not necessarily because of its intrinsic properties, but simply because we value (or ought to value) it for its own sake, that is, not merely as a means. Korsgaard offers the example of a mink coat.

A mink coat can be valued the way we value things for their own sakes: a person might put it on a list of the things he always wanted, or aspire to have some day, right alongside adventure, travel, or peace of mind. Yet it is also odd to say it is valued simply for its own sake. A coat is essentially instrumental: were it not for the ways in which human beings respond to cold, we would not care about them or ever think about them. To say the coat is intrinsically or unconditionally valuable is absurd: its value is dependent upon an enormously complicated set of conditions, physiological, economic, and symbolic.… Mink coats and handsome china and gorgeously enamelled frying pans are all things that human beings might choose partly for their own sakes under the condition of their instrumentality: that is, given the role such things play in our lives. (185)

Perhaps self‐consciousness is like the mink coat. Given a background level of cognitive sophistication as well as phenomenal consciousness, perhaps self‐consciousness can be valued for its own sake.

Such a possibility suggests an isolation test different from, and less obscure than, the one conducted above. If self‐consciousness might be valuable partly for its own sake under the condition of its instrumentality, we might consider two entities with fairly sophisticated mental lives. Let us say that both entities possess good working memory capacity, retain a fair amount of information in short‐ and long‐term memory, have subtle attentional capacities (e.g., abilities to direct covert attention, to focus attention toward different perceptual modalities, and so on), fairly high‐resolution perceptual capacities, abilities to imaginatively simulate a range of counterfactual scenarios, and a flexible behavioral repertoire. Suppose as well that these entities are phenomenally conscious. As before, the only difference between these two entities is that one is self‐conscious. It can token mental states with self‐referring content. The questions to ask concern whether we ought to ascribe more final value to the self‐conscious entity, and whether there are strong reasons against harming or killing the self‐conscious creature that are not present in the case of the non‐self‐conscious entity.

I suspect some people will say that they do value their capacity to think of themselves as themselves, and of features of themselves as features of themselves, for its own sake.^7^ But does this fact support *Self‐consciousness's significance*? The kind of value that accrues to self‐consciousness here seems analogous to the value we might give to a range of mental capacities, such as the capacity to direct covert attention, or generate mental imagery. These capacities are nice to have. But their value seems contingent on predilections of the valuer. (One is not deluded if one fails to value the possession of covert attention, or the capacity to generate mental imagery.) The final value such mental capacities possess thus does not seem sufficient or even necessary for the generation of strong reasons against harming or killing entities that possess them. Analogously, whatever final moral significance self‐consciousness might have does not seem sufficient or even necessary for the generation of strong reasons against harming or killing an entity.

## 4 Conclusion

We want to understand the nature and grounds of the moral significance of entities like (and unlike) us. It is harder to do this if we are distracted by red herrings. In my view, self‐consciousness can and often does function as a particularly shiny red herring. (Why this is so is not something I have speculated on here.) I have considered two ways to defend the claim that the possession of self‐consciousness generates strong moral reasons against harming or killing: one indirect, the other direct. I argued that the best‐known arguments along the indirect route depend in various ways on unwarranted assumptions about self‐consciousness's functional significance and that once these assumptions are undermined motivation for these arguments dissipates. Further, I have considered the possibility that self‐consciousness generates strong moral reasons against harming or killing in virtue of its intrinsic or final moral significance. But it seems that self‐consciousness lacks enough intrinsic or final significance to generate strong moral reasons. We lack good reasons to think that self‐consciousness is highly morally significant. Reflection on the nature and grounds of human and non‐human moral significance should turn elsewhere.^8^

## Notes

1 Three comments. First, I do not wish to deny that self‐consciousness might have some minimal moral significance, nor that self‐consciousness might come to have value for some that possess it. I am concerned with the stronger view that self‐consciousness highly and non‐contingently significant. Second, my focus in this paper is on self‐consciousness's significance for moral patiency. Some maintain that self‐consciousness is significant for moral agency (e.g., Korsgaard 2009)—but that is a subject for another paper. Third, I do not wish to deny that self‐consciousness is necessary for the formation of some desires or goals (desires or goals with self‐referring content). Since it is plausible that some harms are tied to our interests, self‐consciousness might be thought important for the generation of reasons not to carry out particular harms—harms associated with self‐referring desires or goals. In such cases, though, I would insist that analogous desires or goals that lack self‐referring content could be inserted, and thus that the normative role of self‐consciousness here is relatively unimportant.

2 Grouping them together in this way is a somewhat unwieldy move, since there are different ways self‐consciousness could be ‘critical for’ the possession of morally significant properties or capacities. For example, self‐consciousness might ground this possession, or enable it, or partially constitute it, or cause it.

3 In my view, this argument's reliance on future orientation is problematic. The older one gets, the less future‐oriented one might become. It does not become better to kill the aged because they are less future‐oriented than the young, however. Incidentally, this point is recognized by Jeff McMahan (2002) and forms part of the motivation for his two‐tiered account of killing's wrongness. One reason I do not discuss McMahan's account here is that, although McMahan has a place for self‐consciousness in his account, ultimately (as I read him) McMahan's account gives high moral significance to a range of functional properties. For McMahan, what is primarily of moral significance here is ‘possession of certain higher psychological capacities that distinguish [persons] from animals’ (2002: 243).

4 There is a further issue here that I relegate to this footnote. In short, metaphysical personhood is not moral personhood—if we want metaphysical personhood to generate strong moral reasons against harming or killing, we need some kind of bridge principle linking metaphysical personhood and moral significance. Baker appears to endorse such a bridge principle. She asserts, for example, that ‘there can be no ‘right to life’ until there is a person to be a subject of that right’ (2005: 45), where the kind of personhood in question is metaphysical personhood. Should we accept this bridge principle? Notably, Baker offers no argument for the assertion quoted above. And I can see no good reason to accept it. It is not analytic that the only things capable of possessing a right to life are things that qualify as metaphysical persons. Until we know more about the content of metaphysical personhood, it would be wise to withhold judgment about whether metaphysical personhood is necessary for possession of a right to life (or possession of moral significance more generally). And this raises the stakes for what an account of metaphysical personhood should contain if it is to offer an indirect route to moral significance. Not just any account will do. And as we have already seen, Baker's will not.

5 There are of course shades of Moore's (1903) method of isolation here, but the procedure is quite general, and need not involve considering a universe with only one thing in it.

6 A referee notes that many assume self‐consciousness requires phenomenal consciousness. It is important to observe, however, that self‐consciousness (capacity) is simply a kind of cognitive capacity, and as such does not entail or require the possession of phenomenal consciousness. This observation is important not least because the moral significance of phenomenal consciousness should not be conflated with the question at issue here.

7 I might remind such people that while this capacity is fairly interesting, it does not on its own guarantee the transparency of the self to the user of this capacity. Possession of this capacity is consistent with high levels of self‐deception, and even with views according to which there is no self.

8 The author would like to thank Walter Sinnott‐Armstrong and an anonymous referee for excellent comments, as well as audiences at Oxford and Duke University. This work was supported by the Wellcome Trust [104347].
